# Supplementary material for: Development of Comprehensive Serological Techniques for Sensitive, Quantitative and Rapid Detection of Soybean mosaic virus
Source: Int J Mol Sci. 2022 Aug 21;23(16):9457. doi: 10.3390/ijms23169457 (PMC9409097; doi:10.3390/ijms23169457)
Supplement: Supplementary file 1 [file ijms-23-09457-s001.zip › supplementary materials 1.pdf]

Table S1 Relevant Potyvirus strains/isolates analyzed in this study

Strain or isolate	Accession no.	Genome length(nt)	Isolated region	Origin host
4278-1 (SC7)	KT285170.1	9994	China	Glycine max
4469-4 (SC7)	HM590055.1	9994	China:	Glycine max
6067-1 (SC15)	JF833015.1	9588	China: Guangxi	Glycine max
6202-2 (SC21)	JF833014.1	9589	China:	Glycine max
Aa	AB100442	9588	Japan	Glycine max
Aa15-M2	AB100443.1	9588	Japan	Glycine max
Ar13	KF135488.1	9587	Iran	Glycine max
Ar33	KF297335.1	9584	Iran	Glycine max
BCMNV-NL8	HQ229994	9627	USA	Phaseolus
BCMV-Y	AJ312438	10062	China:	Cowpea
CABMV-MG-Avr	AF348210	9465	Zimbabwe	Cowpea
CLLV-BM19	EF105298	9723	Taiwan	Calla lily
G1	FJ640977.1	9588	USA	Glycine max
G2	S42280.1	9588	USA	Glycine max
G3	FJ640978.1	9588	USA	Glycine max
G4	FJ640979.1	9585	USA	Glycine max
G5	AY294044.1	9588	USA	Glycine max
G5H	FJ807701.1	9588	USA	Glycine max
G6	FJ640980.1	9585	USA	Glycine max
G6H	FJ640981.1	9585	USA	Glycine max
G7	AY216010.1	9588	USA	Glycine max
G7a	FJ640982.1	9588	USA	Glycine max
G7d	AY216987.1	9588	USA	Glycine max
G7H	AY294045.1	9588	South Korea	Glycine max
Go11	KF135491.1	9584	Iran	Glycine max
India	KM979229.1	9585	India	Glycine max
L	EU871724	9585	Canada	Glycine max
Lo3	KF135490.1	9584	Iran	Glycine max
L-RB	EU871725	9585	Canada	Glycine max
N	D00507.2	9588	USA	Glycine max
NP-C-L	HQ166265	9617	Canada	Glycine max
NP-L	HQ166266	9617	Canada	Glycine max
Rsv4-RB3	JN416770	9617	Canada	Glycine max
SC3	JF833013.1	9589	China	Glycine max
SC6	HM590054.1	9589	China: Jiangsu	Glycine max
SC6-N	KP710867.1	9589	China	Glycine max
SC7-N	KP710868.1	9994	China	Glycine max
SX-Z	KP710870.1	9587	China:	Glycine max
WMV-Fr	AY437609	10035	France	Panax ginseng
WS149	FJ640968.1	9585	South Korea	Wild soybean
WS160	FJ640972.1	9585	South Korea	Wild soybean
WS200	FJ548849.1	9585	South Korea	Wild soybean
WS209	FJ640976.1	9585	South Korea	Wild soybean
WS32	FJ640954.1	9585	South Korea	Wild soybean
WS37	FJ640955.1	9585	South Korea	Wild soybean
WVMV-Beijing	AY656816	9695	China: Beijing	Wisteria
XFQ001	KP710871.1	9588	China:	Glycine max

Table S2 Primer sequences used in this study

**Primer name**	**Primer sequences (5' to 3')**
SMV-*cp*-F	TCAGGCAAAGAGAAGGAAG
SMV-*cp*-R	TTACTGCTGTGGGCCCATGCCCAA
pCZN1-SMV-*cp*-F	GGCATATGTCAGGCAAAGAGAAGGAAG
pCZN1-SMV-*cp*-R	GCGGATCCTTACTGCTGTGGGCCCATGCCCAA
SMV-*cp*-RT-F	TCAGGCAAGGAGAAGGAAGG
SMV-*cp*-RT-R	GCAAGTGGTCCAAACTGAGAAT
Q-*GmTubulin*-F	GGAGTTCACAGAGGCAGAG
Q-*GmTubulin*-R	CACTTACGCATCACATAGCA

Note: The two *Nde* I and *Xba* I restriction endonuclease sites (underlined sequences) were arranged to the 5’-end of the forward and reverse primers.

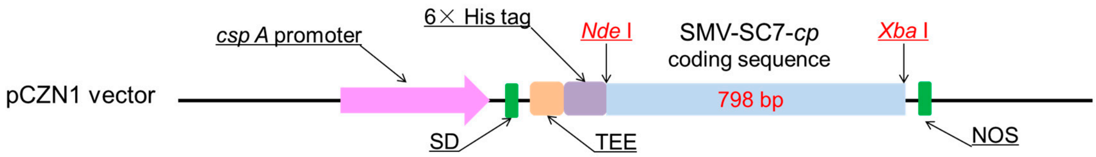

Fig. S1. Schematic of recombined vector pCZN1-SMV-*cp*.

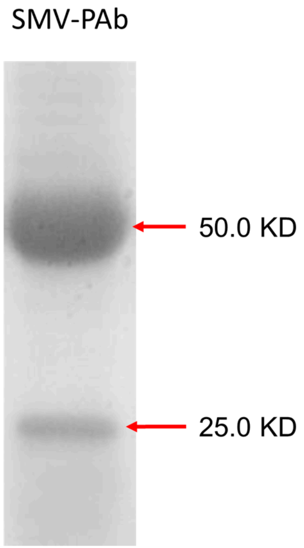

Fig. S2. SDS-PAGE and Coomassie blue staining showed that the purity of the purified antibody.

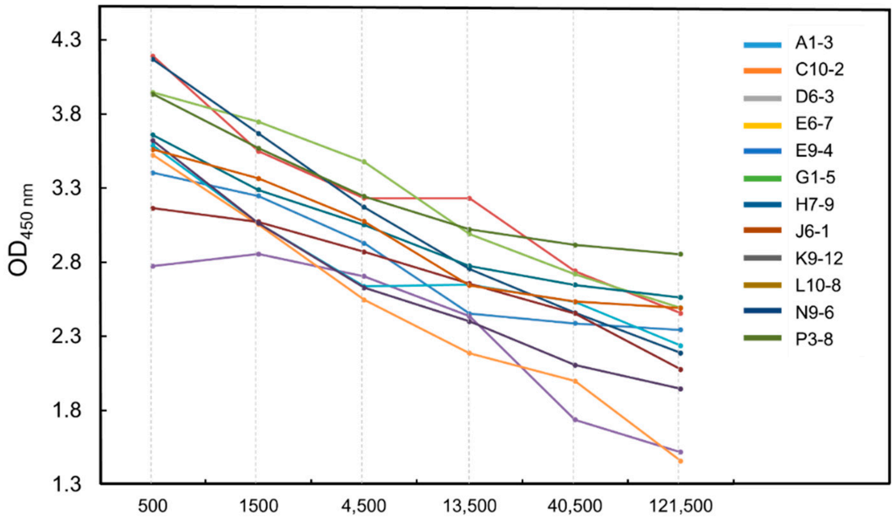

Fig. S3. Supernatants from 12 clones were screened and SMV was recognized by indirect ELISA.
